# Agonists Specific for κ-Opioid Receptor Induces Apoptosis of HCC Cells Through Enhanced Endoplasmic Reticulum Stress

**DOI:** 10.3389/fonc.2022.844214

**Published:** 2022-03-31

**Authors:** Mengyuan Tan, Hanyu Wang, Cheng Gao, Zhen Jiang, Ying Yin, Ruyi Xing, Ling Hu, Jiegou Xu, Min Zhang, Yanhu Xie

**Affiliations:** ^1^ Department of Anesthesiology, The First Affiliated Hospital of USTC, Division of Life Sciences and Medicine, University of Science and Technology of China, Hefei, China; ^2^ Department of Immunology, Anhui Medical University, Hefei, China

**Keywords:** opioid receptors, opioid agonists, hepatocellular carcinoma, apoptosis, PERK pathway

## Abstract

Cancer pain is an important factor affecting life quality of patients especially in the advanced stage and relieving pain is one of fundamental strategies for cancer treatment. Opioids such as morphine are the most widely used in clinics. However, they have been reported to be associated with the occurrence and development of several types of cancer. Thus, search for an opioid that has analgesic effect and can retard cancer progress simultaneously is critical for cancer management. In this study, we first examined the expression of μ and κ (MOR and KOR) in cell lines and tumor tissues of hepatocellular carcinoma (HCC), a malignant tumor with high mortality, and then compared the effects of opioid receptors-specific agonists on malignant phenotypes of HCC cells *in vitro* and tumor growth in an HCC xenograft mouse model. KOR and MOR were found to be highly expressed in HCC cell lines and HCC tissues. The KOR-specific agonist U50488h, oxycodone (agonist for both KOR and MOR) and the MOR-specific agonist morphine inhibited HCC cell proliferation, while only U50488h and oxycodone suppressed colony formation and migration of HCC cells. U50488h and oxycodone, but not morphine, induced HCC apoptosis. Further detection of PERK, GRP78 and CHOP revealed that PERK signaling was upregulated by treatment with U50488h, while treatment with the PERK inhibitor GSK2656157 partially reversed the promotion of apoptosis and inhibition of cell proliferation by U50488h, indicating that endoplasmic reticulum stress is associated with its suppressing effect on HCC malignant phenotypes. Similar to the *in vitro* results, HCC growth was significantly reduced by administration of U50488h and oxycodone, but not by morphine, in the HCC xenograft mouse model. PERK and caspase-3 in the HCC tissues were up-regulated by U50488h treatment as detected by immunohistochemistry and western blotting. Taken together, our results revealed that activation of KOR by U50488h inhibited malignant phenotypes of HCC both *in vitro* and *in vivo*, while activation of MOR by morphine did not have such effect. Because of their dual roles in the relief of pain and in the suppression of malignant phenotypes, opioids such as U50488h that act on KOR should be considered as the first choice for HCC management.

## Introduction

The latest statistical data indicate that hepatocellular carcinoma (HCC) is one of the most common lethal malignancies and represents the sixth leading cancer and the third in terms of cancer-related mortality worldwide (1). A large proportion of patients with advanced HCC suffer from moderate and severe cancer-related pain (2). Cancer-related pain is a major factor affecting the life quality of cancer patients, and alleviating the pain of cancer patients has great significance in the management of cancer. Opioids, especially morphine, are routinely used for preoperative pain management in cancer patients undergoing surgery, and are also recommended as the standard care in cancer patients in terminal phases (3, 4). Despite the undisputed status of opioids in the relief of cancer-related pain, their roles in cancer progression and overall survival remains to be determined (5–8).

Opioids exert their roles mainly *via* their receptors (9).Three major subtypes of opioid receptors (ORs), i.e. μ-OR (MOR), δ-OR (DOR) and κ-OR (KOR), have similar physiological functions in modulation of locomotion, pain perception and emotional behavior (10). According to the clinical evidence, the first choice step III opioid analgesic ladder proposed by World Health Organization are morphine, oxycodone, and hydromorphone (3), and all of them act primarily on MOR. These MOR agonists, the most widely used in clinics, has proven effective in the treatment of cancer-related pain, but their effects on tumor proliferation remain controversial. Overexpression of MOR has been reported to promoted various types of tumor progression (11–13). Similarly, administration of morphine enhances HCC growth and metastasis (14). However, other studies have shown that MOR-1 expression was higher in tumor tissue compared to non-tumor tissue (15) and MOR agonists have ability to suppress cancer progression (16).

KOR is widely expressed in peripheral tissues and the central nervous system and is associated with chronic pain, addiction, and depression (17). While KOR expression seems to be negatively correlated with tumor growth (18, 19), KOR acts as a potential tumor suppressor in HCC and downregulation of the KOR is strongly associated with poor prognosis (20). However, the role and underlying mechanisms of KOR agonists in HCC have been seldom studied both *in vitro* and *in vivo*. At present, however, there are no specific KOR agonists for the treatment of cancer-related pain in clinic.

For these reasons, in order to determine the role of ORs agonists in regulation of tumor growth and proliferation in HCC, the present study was performed on the HCC cells Hep3B and Huh7. Furthermore, we used xenograft model to study the effects of opioid receptor agonists on tumorigenicity of HCC.

## Materials and Methods

### Patient Specimens

HCC and adjacent non-tumorous tissue samples were obtained from The First Affiliated Hospital of University of Science and Technology of China (Hefei, China). The experimental protocol for the use of tissues was approved by the Ethics Committee of The First Affiliated Hospital of University of Science and Technology of China, and written informed consent was obtained from all study participants.

### Cell Lines and Culture

HepG2, Bel 7402, Hep3B and Huh7, human HCC cell lines, were purchased from Procell Life Science & Technology Co., Ltd. The normal hepatocyte, LO2, was acquired from The Cell Bank of Type Culture Collection of The Chinese Academy of Sciences. The cells were cultured at 37˚C in an incubator with 5% CO_2_ in Dulbecco’s Modified Eagle’s Medium (DMEM, Biological Industries, Israel) supplemented with 10% FBS (Biological Industries) and 1% penicillin-streptomycin.

### Drugs and Treatment

U50488 hydrochloride (U50488h, cat. no.0495/25), a specific agonist of KOR, was purchased from Tocris Bioscience Co., Ltd (Bristol, England); morphine Hydrochloride, a specific agonist of MOR, was obtained from Northeast Pharmaceutical Group (Shenyang, China); and oxycodone hydrochloride, an agonist for both KOR and MOR, was from Mengdi Pharmaceutical Co., Ltd. (Beijing, China). GSK2656157 (cat. no. T2654 from Topscience, Shanghai, China), a highly specific and ATP-competitive eukaryotic translation initiation factor 2α (PERK) inhibitor, was dissolved in dimethylsulfoxide (DMSO) to a stock concentration of 2 mM and stored at − 20°C, and diluted 1:1000 in cell culture medium to make a working solution. Hep3B and Huh7 were incubated with U50488h (0,0.1,1,10 μM), oxycodone (0,0.1,1,10 μM) and morphine (0,0.1,1,10 μM) for 24h and 48h in viability assay. The concentration of drugs and the time of stimulation in the follow-up experiments were chosen based upon successful inhibition in viability assay: U50488h (10 μM) oxycodone (10 μM) and morphine (10 μM) for 48h.

### Immunofluorescence (IF)

For immunofluorescence, climbing slides were pre-placed in six-well plate, then LO2, Hep3B, and Huh7 cells (3×10^5^/well) were seeded into the six-well plate, cultured in a 37°C and 5%CO_2_ incubator overnight. 4% paraformaldehyde (biosharp, China) was used to fix at room temperature for 20 minutes and 50ul of blocking solution for immunofluorescence (Proteintech,China) was dropped on the climbing slide and incubated at room temperature for 1 hour. The following primary antibodies KOR (1:100; cat. no. ab113533; Abcam, UK) and MOR (1:100, cat. no.DF5045; Affinity, China) were diluted in the climbing slides, and incubated overnight at 4°C in a wet box. On the next day, the climbing slides were washed three times with phosphate-buffered saline tween-20(PBST), each time for 5 minutes, then 50ul Cy3 Goat anti-rabbit secondary antibody (1:100, Proteintech, China) was added to the climbing slides, and incubated in a cassette at room temperature for 1 hour. The slides were taken out from the six well plate, and a drop of a fluorescent antiquenching agent (meilunbio, China) containing DAPI was placed on the glass slide, covered under a coverslip, avoiding air bubbles. The cells were observed with the fluorescence microscope (Nikon type 120c, Japan).

### Reverse Transcription-Quantitative PCR (RT-qPCR)

Total RNA was extracted from cells using TRIzol^®^ reagent (Invitrogen; Thermo Fisher Scientific, USA). Total RNA was reverse transcribed into cDNA using the HiScript Q RT SuperMix for qPCR (Vazyme Biotech, China) according to the manufacturers’ instructions. qPCR was subsequently performed using specific primers and an AceQ qPCR SYBR Green Master Mix (Vazyme Biotech) with the following cycling parameters: 95°C for 5 min, followed by 40 cycles of 95°C for 10 s and 60°C for 30 s. The specific primer pairs used for the qPCR analyses are indicated in the **Table S1** of the **Supplementary Material**. Relative gene expression was calculated using the 2^-ΔΔCq^ method. GADPH mRNA expression level was used as the internal normalization control.

### Western Blotting

Cells or tissues were lysed in RIPA lysis buffer (Beyotime Institute of Biotechnology) on ice for 30 min and the lysate was centrifuged at 3000g, 4°C for 15 min. The concentration of the proteins in the supernatants was determined using the BCA protein quantification kit (Beyotime Institute of Biotechnology) according to the manufacturer’s instructions. Proteins (30 µg/lane) were separated in 8, 10 or 12% SDS-PAGE, respectively, according to the molecular weight of the target proteins, then transferred onto a PVDF membrane (Millipore, Sigma, USA) using transfer buffer for 60-90 min. The membranes were subsequently blocked with 5% non-fat milk at room temperature for 2 h, and then incubated with the following primary antibodies overnight at 4°C: anti-β-actin (1:5,000; cat. no. AF7018; Affinity Biosciences, USA), anti-KOR (1:900; cat. no. ab113533; Abcam, UK), anti-GRP78 (1:1,000; cat. no. ab191023; Abcam, UK), anti-CHOP (1:500; cat. no. ab11419; Abcam, UK), anti-GADPH(1:8,000; ProteinTech Group, China), anti-caspase-3 (1:3,000; cat. no. 19677-1-AP; ProteinTech Group, China), anti-E-cadherin (1:5,000; cat. no. 20874-1-AP; ProteinTech Group, China), anti-Bax (1:2,000; cat. no. 50599-2-lg; ProteinTech Group, China), anti-PERK (1:1000; cat. no.5683, Cell Signaling Technology, Boston, USA), anti-N-cadherin (1:1,000; cat. no. 13116; Cell Signaling Technology) and anti-vimentin (1:1,000; cat. no. 5741; Cell Signaling Technology). Following the primary antibody incubation, the membranes were washed with TBS-Tween 20 three times and incubated with anti-rabbit (1:10,000; cat. no. A0208; Beyotime Institute of Biotechnology) IgG HRP-conjugated secondary antibodies at 37°C for 2 h. Protein bands were visualized using an ECL kit (Thermo Fisher Scientific, Inc.) and densitometric analysis was performed using ImageJ software (v1.8.0; National Institutes of Health).

### Cell Viability Assay

A total of 6×10^3^ Huh7 and Hep3B cells/well were seeded into 96-well plates and incubated overnight. Following the incubation, the cells were exposed to various concentrations (0, 0.1, 1 and 10 μM) of U50488h, oxycodone or morphine for 24 and 48 h. The cell viability was detected using the cell counting kit-8 (CCK-8, BestBio, Shanghai, China) according to the manufacturer’s protocol. The absorbance was measured using an microplate reader (USCN KIT INC, Wuhan, China). Relative cell viability (%) was calculated using the following equation: (Treated cells – blank well)/(control cells – blank well) x100.

### Colony Formation Assay

For the colony formation assay, 500 cells/well were plated into 6-well plates and cultured with DMEM supplemented with 10% FBS at 37°C in 5% CO_2_ for 2 weeks. The cells were fixed with methanol for 25 min and stained with crystal violet solution for 15 min. Visible colonies were imaged and observed using phase-contrast microscope (Olympus, Japan), and counted using ImageJ software.

### Flow Cytometric Analysis of Apoptosis

Huh7 and Hep3B cells were treated with 10 µM U50488h, 10 µM oxycodone or 10 µM morphine at 37°C for 48 h, and then stained with an Annexin V/FITC Apoptosis Detection kit (BestBio, Shanghai, China) according to the manufacturer’s protocol. The percentage of early and late apoptotic cells was analyzed using a FACSCalibur flow cytometer (Becton–Dickinson, San Jose, CA, USA).

### Wound Healing Assay

The migratory ability of HCC cells was analyzed using a wound healing assay. Briefly, 4◊105 cells were seeded into 6-well plates, cultured to 70% confluence, treated with DMEM, 10 µM U50488h, 10 µM oxycodone or 10 µM morphine and incubated for 48 h at 37°C. The cell monolayer was scratched with a 200ul pipette tip to create an artificial wound, washed with PBS three times, and then incubated with serum-free DMEM at 37°C with 5% CO2 for another 48 h. The width of the wound area was measured at 0 and 48 h after the wound creation. The migratory ability of the cells was observed and calculated as the wound area at 48 h compared with the wound area at 0 h.

### Mouse Xenograft HCC Model

Female 5 to 6-week-old and 20-25g Balb/C nude mice were selected to establish the xenograft model. 5◊10^6^ Hep3B cells were injected into the right axilla of 20 mice. The mice were randomly divided into four groups: PBS (control), U50488h (1.25 mg/kg) (21), oxycodone (0.307mg/kg/d for first 15 days and then 0.715 mg/kg/d, equivalent to the analgesic potency of morphine), and morphine (0.714 mg/kg/d for first 15 days and then 1.43 mg/kg/d) (22). PBS and drug treatments were started on day 5 (the tumor grew to about 30-60mm^3^), intraperitoneally injected every two days. A vernier caliper was used to measure the width (a) and length (b) of the tumors and the tumor volumes were calculated by the formula (a^2^×b)/2. All the mice were sacrificed on the 21st day post Hep3B injection and the tumors were weighed. The animal experiment was approved by the Animal Protection and Use Committee of the First Affiliated Hospital of the University of Science and Technology of China (2021-N(A)-56).

### Histopathological Immunohistochemistry (IHC)

For IHC staining for PERK and caspase3 expression in HCC xenograft in mice, consecutive 4μm sections of the paraffin-embedded tumor specimens were prepared for hematoxylin-eosin (HE) staining and IHC. The sections were deparaffinized in xylene and rehydrated with a descending alcohol series. After antigen retrieval using sodium citrate and endogenous peroxidase activity blocking in 3% hydrogen peroxide at 37°C for 10 min, the tumor tissues were incubated with a rabbit anti-PERK antibody (1:2000; cat. no.5683, Cell Signaling Technology, Boston, USA), or rabbit anti-caspase3 antibody (1:500; cat. no. 19677-1-AP; ProteinTech Group, Wuhan, China) overnight at 4°C. Following the primary antibody incubation, the sections were rinsed with PBS three times (5 min each time) and incubated with an HRP-conjugated goat anti-rabbit secondary antibody (1:200; cat. no. S0001, Affinity, USA) at 37°C for 30 min. After washing, the slides were stained with diaminobenzidine and counterstained with hematoxylin solution. Finally, the tissue sections were mounted and observed under a light microscope. Obvious brownish staining in the cytoplasm were identified as positive cells. Assessment of the mean of integrated optical density (IOD) staining was evaluated using Image-Pro Plus 6.0 software.

### Statistical Analysis

Data are presented as the mean ± SD of at least three independent experiments. Statistical analyses were performed using GraphPad Prism 8.0 software (GraphPad Software, Inc.). A one-way ANOVA was used for multigroup comparisons and Student’s t-test was used to compare the statistical differences between two groups. P<0.05 was considered to be a statistically significant.

## Results

### High Expression of KOR and MOR in HCC Cell Lines and Human HCC Tissues

**Figure 1:**
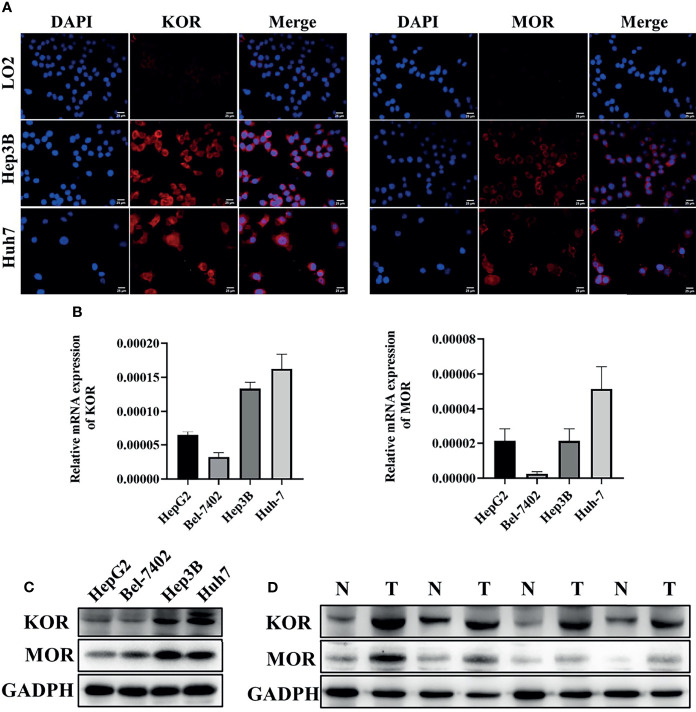
The expression of KOR and MOR in HCC cell lines and human HCC tissues. **(A)** The expression of KOR and MOR were detected in LO2, Hep3B and Huh7 cells by immunofluorescent staining, scale bar, 25um. The expression of KOR and MOR was evaluated in 4 types of HCC cell lines (HepG2, Bel-7402, Hep3B and Huh-7) by RT-qPCR **(B)** and western blotting **(C)**. **(D)** In human HCC tissues, KOR and MOR were detected by western blotting, GADPH was used as an internal control. N, Non-tumor; T, Tumor. Values are presented as the mean ± standard deviation (n = 3). KOR, kappa opioid receptor; MOR, mu opioid receptor; GADPH, glyceraldehyde-3-phosphate dehydrogenase.

For comparison of the expression of KOR and MOR, the normal hepatocyte LO2 and the human HCC cell lines HepG2, Bel-7402, Hep3B and Huh-7 were used. Immunofluorescent staining revealed that KOR and MOR proteins were expressed on the plasma membrane of the HCC cells, the expression level of KOR and MOR were higher in Hep3B and Huh7 cells compared with LO2(**Figure 1A**). RT-qPCR analyses indicated that mRNAs of KOR and MOR were highly expressed in Hep3B and Huh7 (**Figure 1B**). KOR and MOR proteins expression level by western blotting was similar to the RT-qPCR results (**Figure 1C**). So Hep3B and Huh7 were chosen for subsequent experiments. Furthermore, western blotting analysis demonstrated that the expression levels of the KOR and MOR were upregulated in the HCC tissues compared with those in the adjacent non-tumorous tissues (**Figure 1D**).

### Inhibition of Malignant Phenotypes of HCC Cells by KOR Agonists

**Figure 2:**
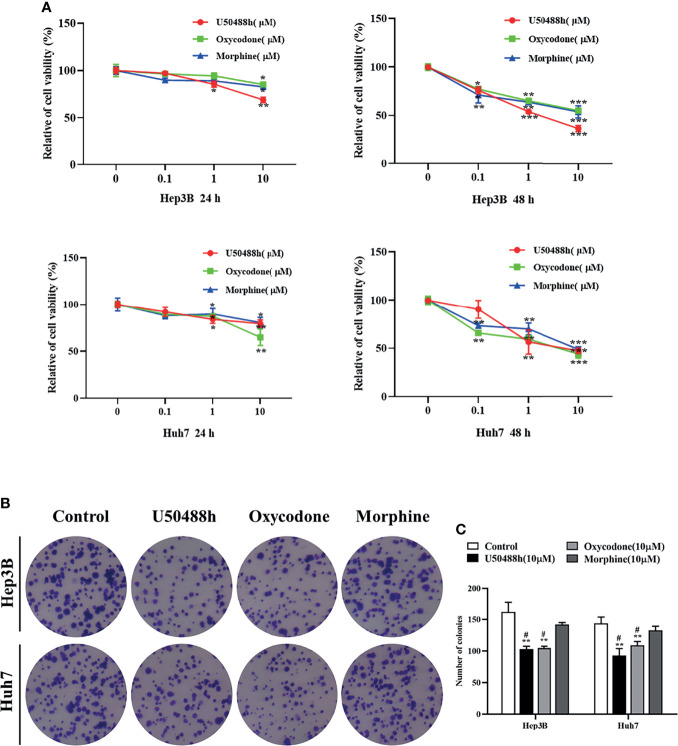
KOR agonist inhibits proliferation in HCC cells. **(A)** Hep3B and Huh7 cells were treated with the KOR agonist U50488h (0,0.1,1, and 10μM), the mixed agonist oxycodone (0,0.1,1, and10μM) and the selective MOR agonist morphine (0,0.1,1, and10 μM) for 24 h and 48 h, and CCK-8 assay was performed to assess cell viability. **(B, C)** Hep3B and Huh7 cells were treated with U50488h (10 μM), Oxycodone (10 μM) and Morphine (10 μM) for 48 h for colony formation assay. Values are presented as the mean ± standard deviation of three independent experiments. *P < 0.05, **P < 0.01, ***P < 0.001 vs. control group; ^#^P < 0.05 vs. Morphine group.

**Figure 3:**
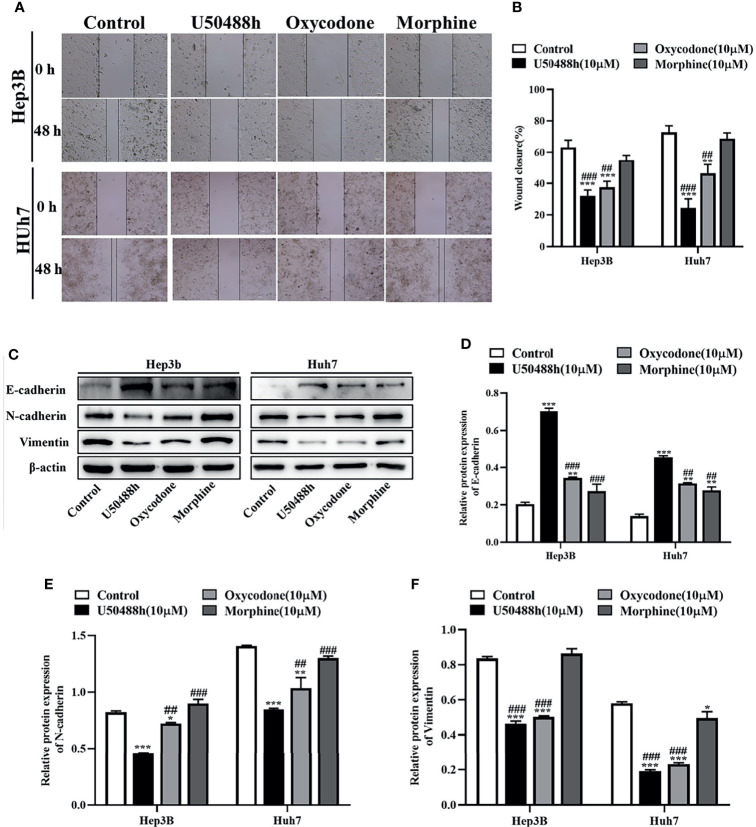
KOR agonist U50488h and oxycodone decreases the migration of HCC cells. **(A)** Hep3B and Huh7 cells were treated with 10 μM U50488h, 10 μM oxycodone and 10μM morphine for 48 h to detected the motility of cells, and wound healing assay was performed, scale bar=100 um. **(B)** Histogram represents the wound closure of cells. **(C-F)** Western blotting was used to detect the expression of E-cadherin, N-cadherin, Vimentin and β-actin in Hep3B and Huh7 cells treated with U50488h (10 μM), oxycodone (10 μM) and morphine (10 μM). β-actin was used as an internal control. Values are reported as the mean ± standard deviation (n = 3). *P < 0.05, **P < 0.01, ***P < 0.001 vs. control group; ^##^P < 0.01, ^###^P < 0.001vs. U50488h group.

Since expression of KOR or MOR was much higher than that of DOR in the HCC tissues and cell lines as described above, agonists specific for KOR and MOR were tested to observe their effects on malignant phenotypes of HCC cells. As shown in the **Figure 2A**, treatment of Hep3B and Huh7 cells with increased concentrations of U50488h (a KOR-specific agonist), oxycodone (an agonist for both KOR and MOR), or morphine (an MOR-specific agonist) for 24 and 48 h reduced cell proliferation in a dose- and time-dependent manner. Furthermore, U50488h and oxycodone significantly suppressed the colony formation of both Hep3B and Huh7 cells (**Figure 2B**). However, the MOR-specific agonist morphine did not exert this effect (**Figures 2B, C**). Similarly, wound healing assay revealed that the migration of HCC cells was inhibited by U50488h or oxycodone to varying degrees, but not by morphine, as compared with cell migration in the control group (**Figures 3A, B**). Consistent with the results of wound healing assay, western blotting analyses revealed upregulated expression of E-cadherin and downregulated expression of N-cadherin and vimentin (**Figures 3C–F**) by treatment with U50488h or oxycodone, indicating that U50488h or oxycodone suppressed the epithelial-mesenchymal transition (EMT) of the HCC cells. Morphine also increased the expression of E-cadherin as compared to the control, but to a less extent than U50488h or oxycodone (**Figures 3C, D**). Since the expression of N-cadherin and vimentin in the morphine-treated cells was comparable to that of the control (**Figures 3C, E, F**), morphine did not seem to have an obvious inhibition of EMT. These results demonstrated that the inhibitory effect on HCC cells migration by U50488h or oxycodone was *via* suppression of EMT. Taken together, malignant phenotypes of HCC cells including cell proliferation, colony formation and migration can be suppressed by KOR activation either *via* the KOR-specific agonist U50488h or *via* the mixed agonist oxycodone, but not by MOR activation *via* the MOR-specific agonist morphine.

### Induction of HCC Cell Apoptosis by KOR Agonists

**Figure 4:**
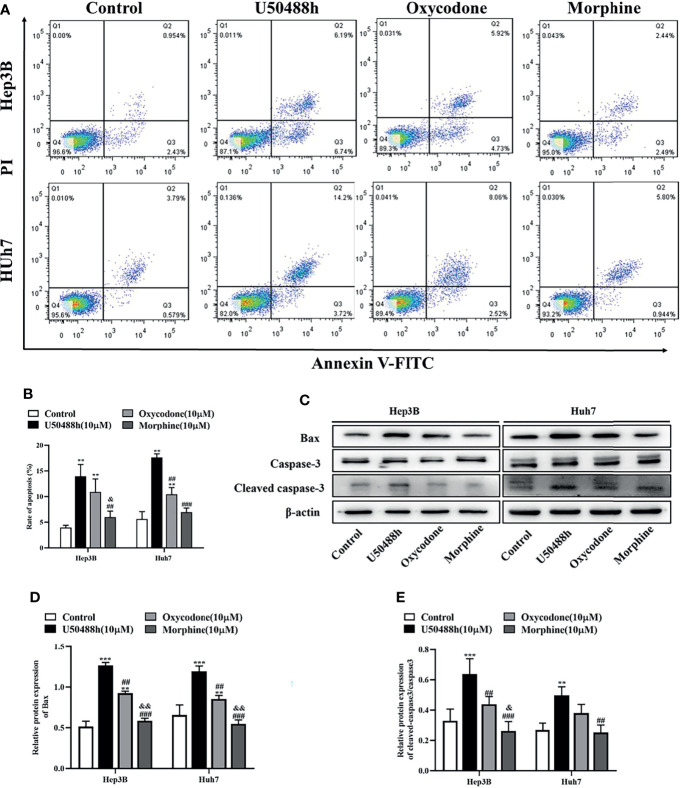
KOR agonist induces apoptosis in HCC cells. **(A)** Hep3B and Huh7 cells were exposed to 10 μM U50488h, 10 μM oxycodone and 10 μM morphine for 48 h using PI/Annexin V-FITC flow cytometry. **(B)** Histogram indicates the rate of apoptosis of cells. **(C–E)** Cells were cultured with 50 μM U50488h, 10 μM oxycodone and 10 μM morphine for 48 h, and the expression of apoptosis-related proteins such as Bax and Cleaved caspase-3 were analyzed by western blotting. β-actin was used as an internal control. Values are reported as the mean ± standard deviation (n = 3). **P < 0.01, ***P < 0.001 vs. control group; ^##^P < 0.01, ^###^P < 0.001 vs. U50488h group; ^&^P < 0.1, ^&&^P < 0.01 vs. oxycodone group. PI, propidium iodide; FITC, fluorescein isothiocyanate.

For comparison of apoptosis-inducing abilities of the KOR and MOR agonists, Hep3B and Huh7 cells were treated with 10μm of U50488h, oxycodone or morphine for 48h, and the apoptotic cells were then analyzed by flow cytometry. As shown in **Figures 4A, B**, a significant increase of apoptosis rate was found in the U50488h- or oxycodone-treated cells compared to the control treatment, but not in the morphine-treated cells. In Hep3 and Huh7 cells, pro-apoptotic proteins Bax and cleaved caspase 3 were increased by U50488h or oxycodone treatment, but not by treatment with morphine (**Figures 4C–E**). These results indicate that HCC apoptosis can be induced by KOR activation, but not by MOR activation.

### Enhanced ER Stress of HCC Cells by U50488h

**Figure 5:**
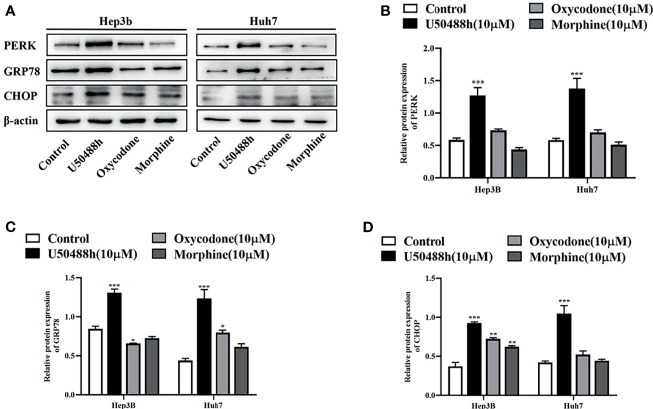
KOR agonist induced endoplasmic reticulum (ER) stress in HCC cells. HCC cell lines Hep3B and Huh7 incubated with U50488h (10 μM), oxycodone (10 μM) and morphine (10 μM) for 48 h, ER stress-related mRNAs and proteins including GRP78, PERK, and CHOP were detected by RT-qPCR (S1) and Western blotting **(A–D)**. β-actin was used as an internal control. Values are reported as the mean ± standard deviation (n = 3). *P < 0.05, **P < 0.01, ***P < 0.001 vs. control group.

For understanding the underlying mechanism of apoptosis induced by the KOR agonists, RT-qPCR was first performed to analyze the relative mRNA expression of endoplasmic reticulum (ER) stress-associated molecules including GRP78, PERK and CHOP. The results revealed that the expression of these molecules was significantly increased by the KOR agonist U50488h compared with those in the control group (**Figure S1**). Similarly, increased protein expression of GRP78, PERK and CHOP in the U50488h-treated cells was detected by western blotting (**Figures 5A–D**). However, the MOR agonist morphine did not induce an increase of GRP78, PERK and CHOP at both the mRNA and protein levels (**Figure S1**, **Figures 5A–D**). These results indicate that the specific KOR agonist U50488h may promote apoptosis of HCC cells by upregulating PERK pathway.

To further explore the relationship between ER stress and apoptosis, the PERK inhibitor GSK2606414 was used. Hep3B and Huh7 cells were co-treated with 2 µM GSK2606414 and the selective KOR agonist U50488h (10 µM) for 48 h, and flow cytometric analysis of apoptosis and cell proliferation assay were performed. As shown in **Figures S2A, B**, GSK2606414 reduced the apoptosis rate induced by U50488h. Also, GSK2606414 mitigated the inhibitory effect of U50488h on HCC cell proliferation (**Figure S2C**). Western blotting verified that the expression of GRP78, PERK and CHOP was decreased by treatment of GSK2606414 (**Figures S2D–G**). Although GSK2606414 was unable to completely reverse the effects of U50488h on HCC apoptosis and cell proliferation, these findings indicated that the effects were partially *via* the up-regulated PERK signaling.

### Decreased HCC Growth by KOR Agonists in a Xenograft Mouse Model

**Figure 6:**
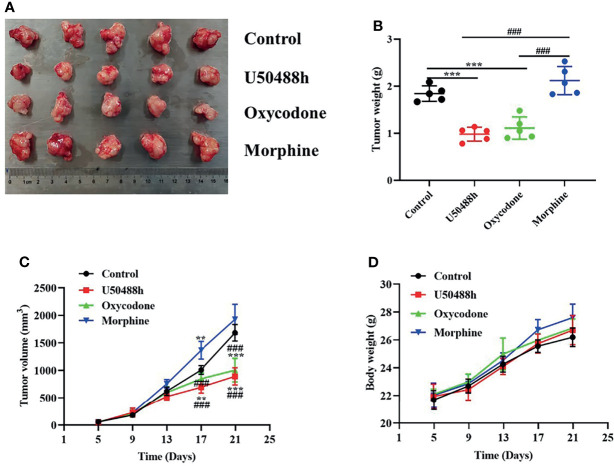
KOR agonist and mixed agonist, oxycodone decreased the tumorigenicity of HCC *in vivo*. **(A)** Representative images of tumors in mice treated with PBS and OR agonists. **(B, C)** Tumor weight and volume of PBS and ORs agonists treated HCC tumors in mice (*n* = 5). **(D)** Body weight growth curves of mice over 21 days following tumor implantation (n = 5). **P < 0.01, ***P < 0.001 vs. control group; ^###^P < 0.001 vs. Morphine group.

**Figure 7:**
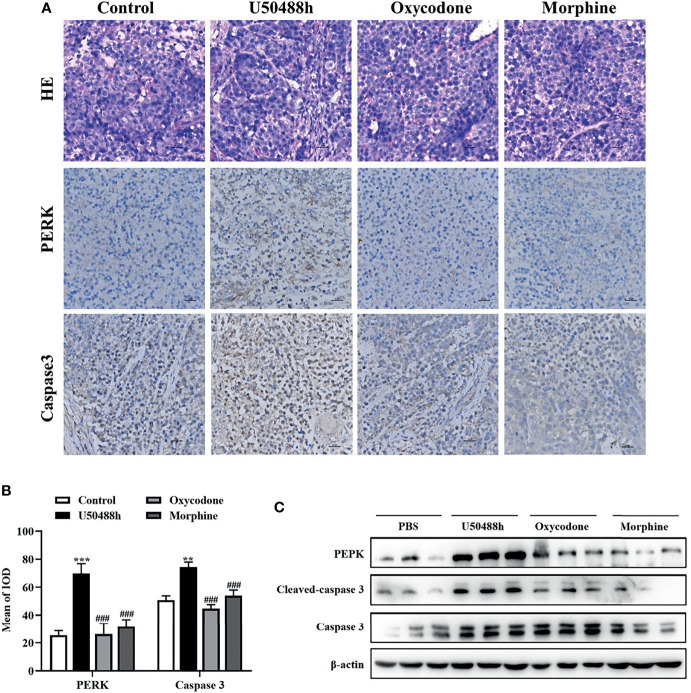
KOR agonist increased expression level of PERK and Caspase 3 *in vivo*. **(A)** Representative IHC staining in tumor tissue for PERK and Caspase 3 in the control and OR agonists treated mice (n = 5) (scaled bar= 25 um); **(B)** Histogram represent the mean of IOD. **(C)** Levels of PERK and Caspase 3 determined by western blot (n = 3). Data are presented as mean ± SEM. **P < 0.01, ***P < 0.001 vs. control group; ^###^P < 0.001 vs. U50488h group.

For observation of the effects of the 3 OR agonists on HCC growth *in vivo*, Balb/C nude mice were injected with 5◊10^6^ Hep3B cells into the right axilla. The drugs (U50488h, oxycodone, or morphine) with equivalent analgesic potency were intraperitoneally injected every two days until the tumor grew to about 30-60mm^3^ and PBS was used as the control. U50488h and oxycodone, but not morphine, reduced the tumor weight and volume compared with the PBS group (**Figures 6A–D**). The OR agonists used in our study did not decrease the body weight of the mice (**Figure 6D**). IHC staining indicated that expression of PERK and caspase 3 was enhanced in the HCC tissues of the mice-treated with U50488h (**Figures 7A, B**), but not with morphine. Similar results were observed in western blotting analysis (**Figure 7C**).

## Discussion

As ORs are found widely distributed on peripheral tissues and the central nervous system (23, 24) the targeting of ORs has long been used as an authorized strategy for treating perioperative pain management, acute and chronic pain and late cancer pain caused by treatment (25, 26). Recently, opioids have been extensively linked to a variety of pro-and anti-tumor properties (9, 27–29). In this study, we investigated the effects of several opioid receptor agonists on the proliferation, apoptosis, migration and other malignant biological behaviors of HCC. The results showed that the cell activity of Hep3B/Huh7 exposed to opioid receptor agonists was inhibited in a concentration-and time-dependent manner. Meanwhile, U50488h and oxycodone could increase the apoptosis rate and inhibit the migration of HCC. In addition, U50488h and oxycodone treatment of HCC cells decreased the tumorigenicity when xenografted in mice.

A number of MOR agonists, such as morphine, have been reported to be effective in alleviating severe pain; however, there were obvious limitations, such as they are prone to abuse, which is associated with an increased, risk of overdose, and serious side effects (30). In addition, several studies on morphine have shown that it promotes the proliferation, apoptosis, migration, and invasion in the breast (22, 31), lung (6, 32, 33) and colorectal cancer cell lines (34), and a few studies indicated that morphine has an anti-tumor effect (35, 36). Its potential mechanisms include the promotion of tumor angiogenesis, cell cycle arrest, and apoptosis of cancer cells (22, 34, 36). Agonists for the KOR, such as nalbuphine, have been shown to provide similar analgesic effects comparable to morphine, suppressing various acute or chronic pain and cancer pain effectively, and reducing morphine-related side effects such as respiratory depression, addiction (37). However, current studies on the relationship between KOR and tumors suggest that KOR may have an anti-tumor effect (19, 20, 38). Our results show that morphine targeting MOR has no anti-tumor effects on HCC cells *in vivo* and *in vitro*, while KOR specific agonist U50488h effectively inhibited the proliferation, migration and apoptosis of HCC cells *in vivo* and *in vitro*, suggesting that KOR may be a potential tumor suppressor gene. Kuzuma N et al. (19) suggest that KOR agonists inhibit the growth of gefitinib-resistant NSCLC cells, which is consistent with our findings. KOR agonists can inhibit the biological behavior of HCC cells and may inhibit tumor progression in our results. Therefore, KOR agonists are more suitable for HCC-related perioperative pain and cancer pain management and the treatment of advanced cancer pain compared to morphine. Oxycodone, an OR agonist dominated by mu and supplemented by kappa, the same as U50488h, represented anti-tumor properties in our results. We think that it may be related to the partial activation of KOR, but further study is still needed.

Previous study has revealed that that KOR may be associated with alternative G protein coupling and the activation of ERK/MAPK-dependent signaling pathways, to modulate analgesic effects (17). However, the underlying mechanism of the KOR-induced modulation of tumor cell biological behavior remains unclear. The ER is an organelle that regulates protein processing, modifications and folding, which ultimately determines cell function, fate and survival (39). There are three known types of transmembrane protein, namely inositol-requiring enzyme 1α, ATF6 and PERK, that function as sensors of ER stress. GRP78, also called BiP, is a molecular chaperone binding-immunoglobulin protein that binds to these sensors and maintains them in the inactive state under normal conditions. However, during ER stress, GRP78 binds to misfolded or unfolded proteins. This event promotes the activation of the sensors and subsequently induces the UPR (40). The abnormal activation of PERK signaling pathway is related to a variety of pathological alterations, it plays an important role in cell proliferation, apoptosis, autophagy, metabolism and many other cellular processes (41, 42). Studies confirm that some drugs induce apoptosis of HCC cells through PERK pathway (43, 44). Thus, we hypothesized that U50488h-induced apoptosis may be associated with the activation of PERK signaling pathway. Our results showed that apoptosis and PERK expression were significantly increased after exposure to kappa-receptor agonist U50488h in Hep3B/Huh7 cells. Then, GSK2656157, a highly specific and ATP-competitive PERK inhibitor, which downregulates PERK expression and inhibits the UPR signaling pathway, was used to verify the relationship between U50488h-induced ER stress and the apoptosis of Hep3B and Huh7 cells. Our results revealed that the effects induced by U50488h were alleviated following PERK inhibitor treatment. These data showed that our hypothesis is reasonable. U50488h-induced apoptosis of HCC cells through upregulating PERK signal pathway, but the underlying mechanism of U50488h regulating PERK remains to be further studied.

Furthermore, mice were treated with clinically relevant doses of opioid receptor agonists to evaluate their effect on tumor growth. The results showed that U50488h and oxycodone treatment had inhibitory effects on the growth of liver cancer transplanted tumor in mice compared to morphine, which was consistent with the effect of opioid receptor agonists on HCC cells *in vitro*. In the follow-up WB and IHC assays, the expression of PERK was markedly upregulated in the U50488h treatment group, which further confirmed our hypothesis.

We are the first to comprehensively compare the effects of two opioid agonists on the biological behavior of hepatocellular carcinoma. Our study is the first to suggest that KOR agonists are able to activate ER stress *via* the PERK signaling pathway. However, it should be acknowledged that we lack in-depth analysis of how KOR upregulates the PERK pathway. Furthermore, additional signaling transduction pathways may be involved in the crosstalk between ER stress and KOR agonists, which should be verified in future studies.

In conclusion, the findings of the present study revealed that treatment with kappa opioid agonists inhibited HCC cells proliferation, migration and promoted apoptosis. The results confirmed that the underlying mechanism of the KOR agonist U50488h-induced apoptosis of HCC cell was *via* PERK signaling pathway. Furthermore, animal experiments suggest that U50488h treatment could reduce the tumorigenicity of hepatocellular carcinoma. Thus, our findings provide a novel strategy for the use of opioids in patients with HCC who suffer from cancer-related pain.

## Data Availability Statement

The raw data supporting the conclusions of this article will be made available by the authors, without undue reservation.

## Ethics Statement

The animal study was reviewed and approved by the Animal Protection and Use Committee of the First Affiliated Hospital of the University of Science and Technology of China (2021-N(A)-56).

## Author Contributions

YX, MZ, JX, YY, and LH conceived and designed the experiments. MT, ZJ, RX, and CG participated in the experiments and the data analysis. MT and HW wrote the manuscript. YX, MZ, and JX were responsible for manuscript revision and confirm the authenticity of all the data. All authors have read and approved the final manuscripts.

## Funding

The present study was supported by the Xiaoping Chen Foundation for the Development of Science and Technology of Hubei Province (CXPJJH118000017-02-03).

## Conflict of Interest

The authors declare that the research was conducted in the absence of any commercial or financial relationships that could be construed as a potential conflict of interest.

## Publisher’s Note

All claims expressed in this article are solely those of the authors and do not necessarily represent those of their affiliated organizations, or those of the publisher, the editors and the reviewers. Any product that may be evaluated in this article, or claim that may be made by its manufacturer, is not guaranteed or endorsed by the publisher.
