# Erratum: Agonists Specific for κ-Opioid Receptor Induces Apoptosis of HCC Cells Through Enhanced Endoplasmic Reticulum Stress

**DOI:** 10.3389/fonc.2022.937625

**Published:** 2022-05-30

**Authors:** Frontiers Production Office

**Affiliations:** Frontiers Media SA, Lausanne, Switzerland

**Keywords:** opioid receptors, opioid agonists, hepatocellular carcinoma, apoptosis, PERK pathway

Due to a production error, there was an error in affiliation 1. Instead of “Division of Life Sciences and Medicine, Department of Anesthesiology, The First Affiliated Hospital of USTC, University of Science and Technology of China, Hefei, China”, it should be “Department of Anesthesiology, The First Affiliated Hospital of USTC, Division of Life Sciences and Medicine, University of Science and Technology of China, Hefei, China”.

The publisher apologizes for this mistake.

The original version of this article has been updated.

