# Supplementary material for: Agonists Specific for κ-Opioid Receptor Induces Apoptosis of HCC Cells Through Enhanced Endoplasmic Reticulum Stress
Source: Front Oncol. 2022 Mar 31;12:844214. doi: 10.3389/fonc.2022.844214 (PMC9008754; doi:10.3389/fonc.2022.844214)
Supplement: Supplementary file 1 [file DataSheet_1.docx]

**Activation of κ-opioid receptor induces apoptosis of HCC cells through enhanced endoplasmic reticulum stress**

Mengyuan Tan, Hanyu Wang, Cheng Gao, Zhen Jiang, Ying Yin, Ruyi Xing, Ling Hu, Min Zhang Jiegou Xu, and Yanhu Xie,

This supplementary material contains xx tables and xx figures.

Table S1: primer pairs;

Figure S1: Hep3B and Huh7 cells were treated with U50488h, oxycodone, morphine for 48 h and the levels of the indicated mRNAs were determined by RT-qPCR.

Figure S2: KOR agonist-induced apoptosis was decreased by upregulating PERK pathway.

**Table S1 Primer pairs used in qPCR analyses**

| **Genes** | **Forward (**5’→3’) | **Reverse (**5’→3’) |
| --- | --- | --- |
| **PERK** | CTCACAGGCAAAGGAAGGAG | AACAACTCCAAAGCCACCAC |
| **GRP78** | GTCCTATGTCGCCTTCACTC | ACAGACGGGTCATTCCAC |
| **CHOP** | CGCCTGACCAGGGAAGTAGA | TGATGCTCCCAATTGTTCATG |
| **GADPH** | CAGGAGGCATTGCTGATGAT | CAGGAGGCATTGCTGATGAT |

PERK: eukaryotic translation initiation factor 2α; GRP78: human heat shock protein family A (Hsp70) member 5; CHOP: human C/EBP-Homologous Protein;


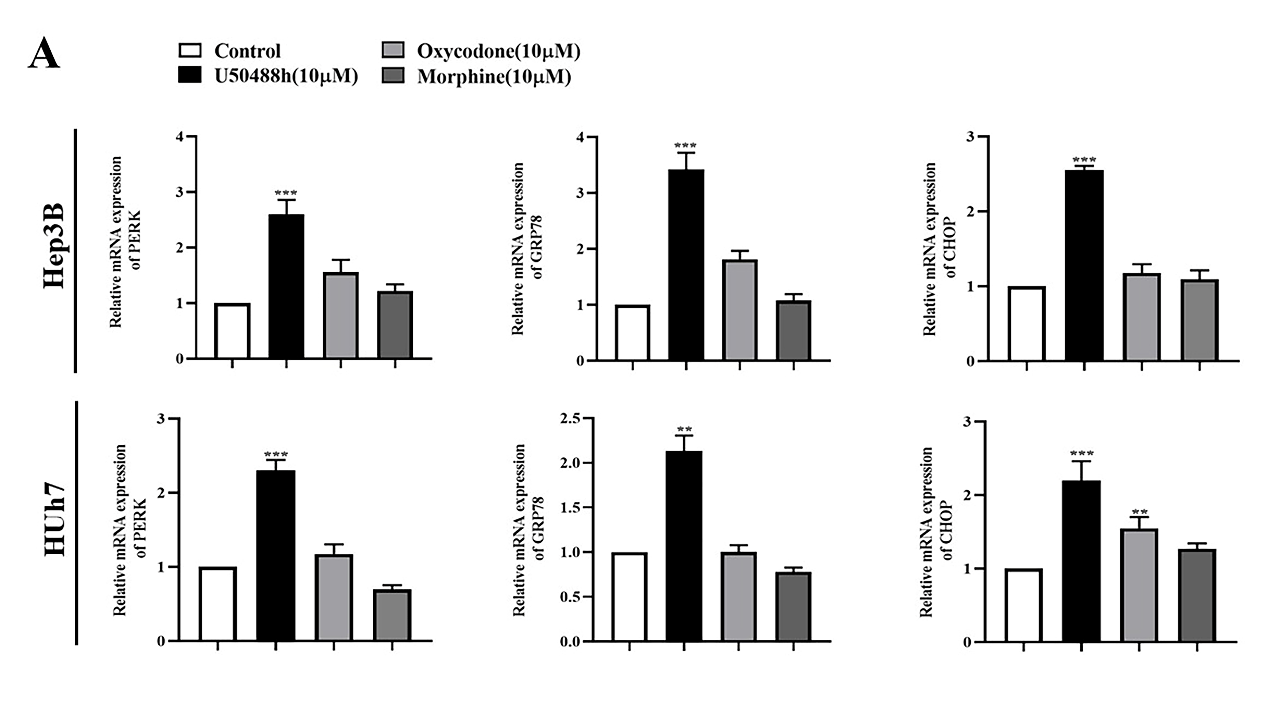


**Supplementary Figure 1.** Hep3B and Huh7 cells were treated with U50488h, oxycodone, morphine for 48 h and the levels of the indicated mRNAs were determined byRT-qPCR.


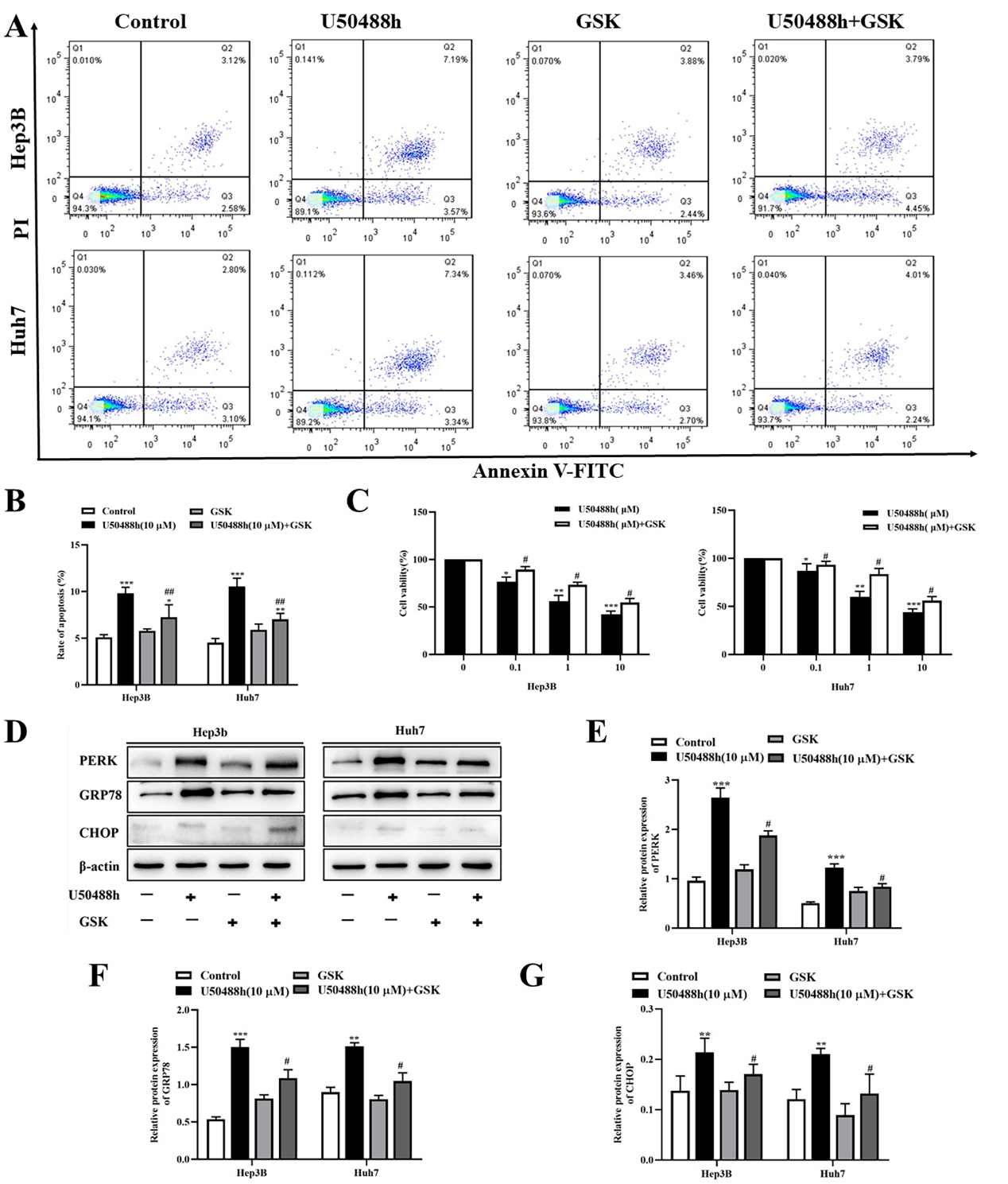


**Supplementary Figure 2.** KOR agonist-induced apoptosis was decreased by upregulating PERK pathway. (A) Hep3B and Huh7 cells cultured with U50488h (10 μM) and/or GSK (2 μM) for 48 h were analyzed by PI/ Annexin V-FITC flow cytometry. (B) Histogram represent the rate of apoptotic cells. (C) Hep3B and Huh7 cells cotreated with different concentration U50488h (0,0.1,1, and 10 μM) and GSK (2 μM) for 48 h, and CCK-8 assay was performed to assess cell viability. (D-G) Hep3B and Huh7 cells were treated with U50488h (10 μM) and/or GSK (2 μM) for 48 h, and ER stress-related proteins including GRP78, PERK and CHOP were analyzed by western blotting. β-actin was used as an internal control. Values are reported as the mean ± standard deviation (n=3). *P<0.05, **P<0.01, ***P<0.001 vs. control group; #P<0.05 vs. U50488h group.
